# Research gaps for three main tropical diseases in the People’s Republic of China

**DOI:** 10.1186/2049-9957-2-15

**Published:** 2013-07-29

**Authors:** Qi Zheng, Samantha Vanderslott, Bin Jiang, Li-Li Xu, Cong-Shan Liu, Le-Le Huo, Li-Ping Duan, Ning-Bo Wu, Shi-Zhu Li, Zhi-Gui Xia, Wei-Ping Wu, Wei Hu, Hao-Bing Zhang

**Affiliations:** 1 National Institute of Parasitic Disease, Chinese Center for Disease Control and Prevention, WHO Collaborating Centre on Malaria, Schisostomiasis and Filariasis, Key Laboratory of Parasite and Vector Biology, Ministry of Health, 207 Rui Jin Er Rd, Shanghai 200025, People’s Republic of China; 2 The Department of Science and Technology Studies, University College London, London WC1E 6BT, UK

**Keywords:** Schistosomiasis, Malaria, Echinococcosis, Epidemiology, Diagnosis, Chemotherapy, Research capacity building

## Abstract

This scoping review analyzes the research gaps of three diseases: schistosomiasis japonica, malaria and echinococcosis. Based on available data in the P.R. China, we highlight the gaps between control capacity and prevalence levels, and between diagnostic/drug development and population need for treatment at different stages of the national control programme. After reviewing the literature from 848 original studies and consultations with experts in the field, the gaps were identified as follows. Firstly, the malaria research gaps include (i) deficiency of active testing in the public community and no appropriate technique to evaluate elimination, (ii) lack of sensitive diagnostic tools for asymptomatic patients, (iii) lack of safe drugs for mass administration. Secondly, gaps in research of schistosomiasis include (i) incongruent policy in the implementation of integrated control strategy for schistosomiasis, (ii) lack of effective tools for *Oncomelania* sp. snail control, (iii) lack of a more sensitive and cheaper diagnostic test for large population samples, (iv) lack of new drugs in addition to praziquantel. Thirdly, gaps in research of echinococcosis include (i) low capacity in field epidemiology studies, (ii) lack of sanitation improvement studies in epidemic areas, (iii) lack of a sensitivity test for early diagnosis, (iv) lack of more effective drugs for short-term treatment. We believe these three diseases can eventually be eliminated in mainland China if all the research gaps are abridged in a short period of time.

## Multilingual abstracts

Please see Additional file 1 for translations of the abstract into the six official working languages of the United Nations.

## Background

Schistosomiasis, malaria and echinococcosis are three types of tropical diseases that threaten more than two billion people worldwide [1-3]. These diseases mostly affect poor rural communities in developing countries [4] and those who are infected with such pathogens not only suffer indisposition but also have various degrees of morbidity, which induce poorer standards of living [5-9].

Continuous economic growth over the last 30 years has allowed the government of the People’s Republic of China (P.R. China) to continuously increased its budget for parasitic disease control, resulting in a significant reduction in disease burden and transmission capacity throughout the country [10-12]. Furthermore, an effective national strategy has successfully brought down the prevalence levels of schistosomiasis japonica and malaria compared to levels of 50 years ago [13-15]. Echinococcosis has also been controlled to a stable level according to the five-year national surveillance report made by the Ministry of Health (MOH). Such achievements can be attributed to political commitments, control strategies adapted to the national control programme, as well as the innovative research and research capacity building [16].

**Figure 1:**
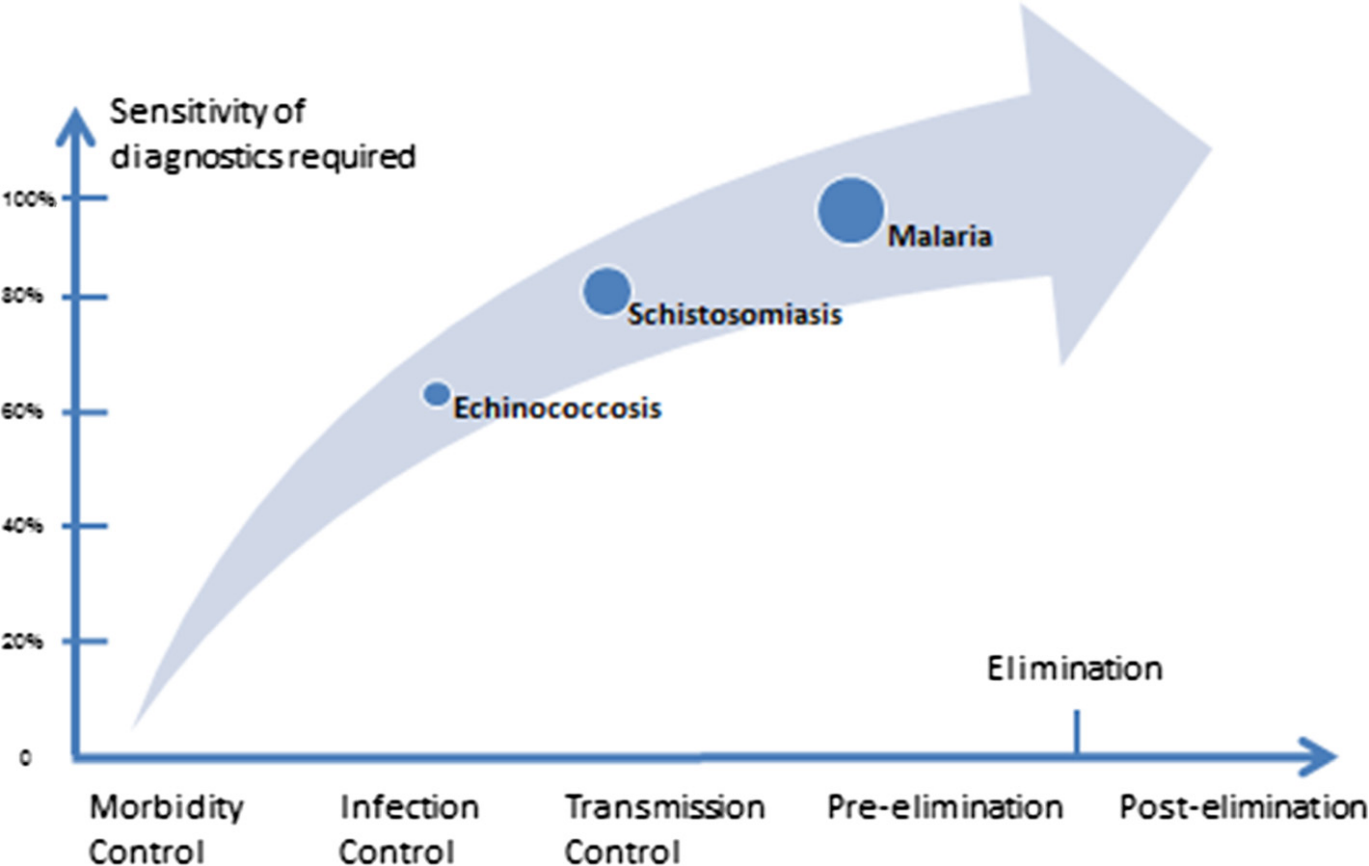
Different stages of the national control programme for three parasitic diseases in China.

The major research achievements of last 30 years are reflected in the progress in drug development, evaluation of diagnostics with the national control programme and the formation of a strong team for operational research, providing the required information and tools for the national control programmes. Due to these diseases being at various stages of the national control programme the sensitivity of diagnosis is also different (See Figure 1). It is believed that without advances in operational research, it will be an uphill struggle to reach the aim of elimination for schistosomiasis japonica and malaria in P.R. China. Moreover, without a new strategy and technical support, the currently controlled situation for echinococcosis may worsen.

## Review

The main purpose of this paper is to summarize and disseminate relevant research findings on the three parasitic diseases, so as to identify research gaps in the existing literature. A consultation study was undertaken through the platform of Chinese Network on Drug and Diagnostic Innovation (China NDI, http://www.chinandi.org.cn) focusing on gaps (i) between research capacity and prevalence, and (ii) between diagnostic/drug R&D capacity and population need for treatment at different stages of the national control programme.

## Methods

Our scoping review uses an adapted version of the ARKSEY and O’MALLEY (2005) framework [17], involving the following steps:

1. Identification and development of the research questions.

The overarching research questions are as follows: What is the gap between control capacity and prevalence among these three diseases? What is the gap between diagnostic/drug R&D capacity and population needs for treatment at the different stages of the national control programmes?

2. Location, screening and selection of relevant publications.

Terms were searched for in two most common databases:PubMed database (http://www.pubmed.com) and international articles from Medline database, as well as the Wanfang database (http://www.wanfangdata.com.cn) for Chinese articles. Cqvip database, Cnki database and Wanfang database are the three most popular Chinese periodical databases in China. The percentage of overlap in core journals of these three databases is more than 95% in the research areas relevant to this scoping review (biotechnology, medicine and health). We selected the Wanfang database after comparing the quality of information by testing key words among three databases mentioned above^a^.

3. Publication selection

The searching procedure yielded 10,835 abstracts. A team constituting of eight professional researchers were responsible for publication selection. Level 1 relevancy testing went as follows:

1) Title, author and abstracts were scanned to determine whether they were relevant with epidemiology, diagnosis and chemotherapy of the three parasitic diseases we focused on in China.

2) The study must have been launched in China and include at least one Chinese author.

**Figure 2:**
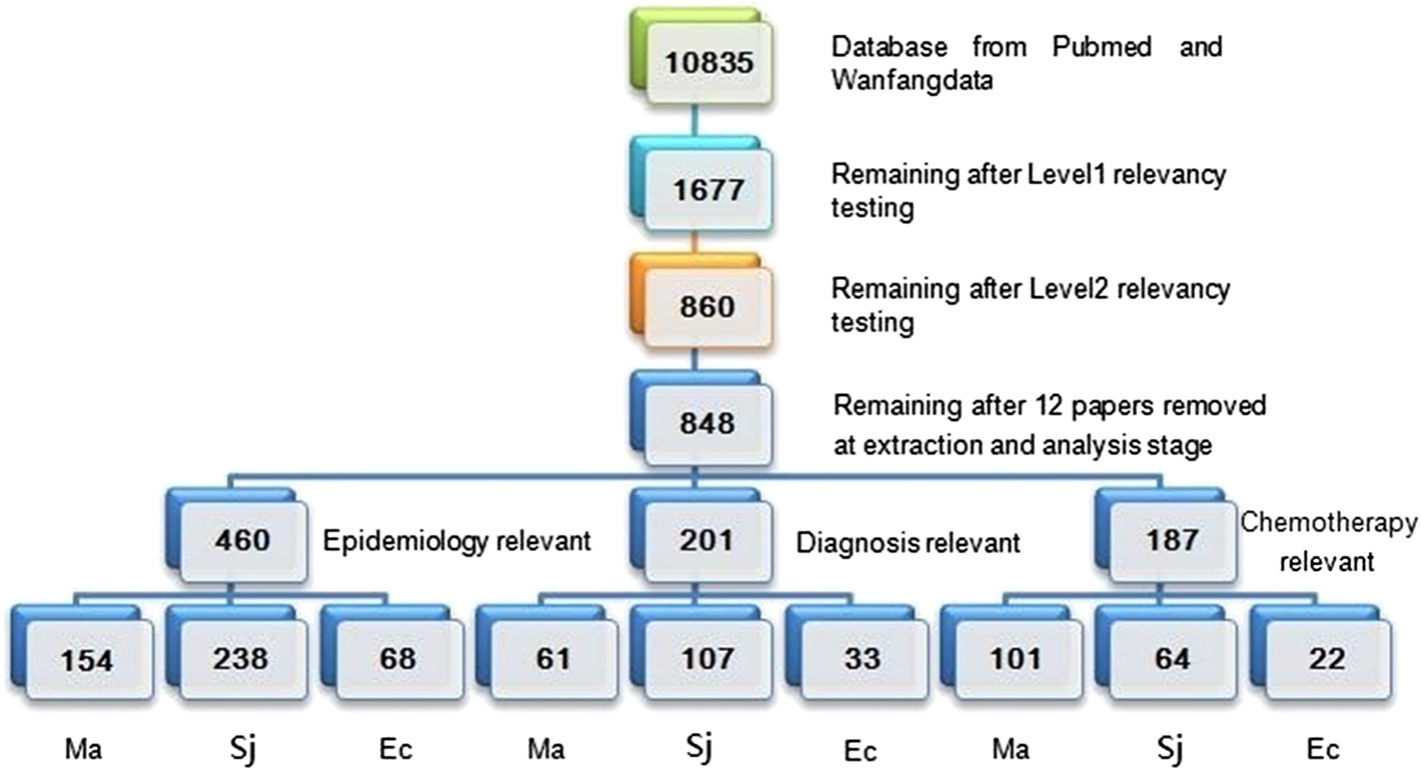
**Results of the search strategy.** (Abbreviation: Ma instead for Malaria; Sj instead for Schistosomiasis japonica; Ec instead for Echinococcosis).

After the level 1 relevancy testing, 1677 citations were identified for inclusion in this paper. Full articles were then obtained for level 2 relevancy testing. Team members selected and eliminated those publications that focused on single case report or treatment, vaccines, animal models, species validation, surgical treatment and phylogenetic study. Through this filtering, 860 articles were deemed relevant and selected for inclusion. Of these articles, 12 were excluded in data extraction as they could not classified into epidemiology, diagnosis and chemotherapy by their full text. A total of 848 articles were included in this research, with the review process is outlined in Figure 2.

4. Data extraction.

The data extracted was consolidated in a ‘data extraction form’ using a database programme, which divided the three different diseases along three research fields (epidemiology, diagnosis and chemotherapy). We collated a mixture of general information about the study along with specific information relevant to our research and recorded the information as follows:

• Author(s), year of publication, location and study site(s)

• Intervention type

• Populations

• Aim

• Methodology

• Outcome measures

• Results

This data formed the basis of the analysis. We sought a uniform approach to all 848 studies included in the review. In practice we found out that approximately 8% of the included articles did not present all the information we needed.

5. Consultation exercise.

As indicated in the background, this scoping study also included a consultation element. Since 2010, more than 300 scholars attended our public meetings to discuss drugs, diagnostics innovation and control for tropical diseases in China. The minutes of these meetings were extracted for gap analysis and considered in comparison to the literature review.

6. Collating, summarizing and reporting the results

Once the outlined steps were completed, we were able to present our narrative account of findings. Particular attention was given to a basic numerical analysis of the studies included in the review. Since there was great diversity and/or overlaps among reports, we rejected much of the very detailed information in order to make the table clearer. We hope these tables can present readers with an intuitive impression of the three diseases in China. In addition the team evaluated the full text of each paper and supplementary information was collated in the corresponding summary table.

## Result

### Gaps between research capacity and disease prevalence

After we extracted and analyzed the chosen articles, the relevant data was collected and summarized for thematic findings from the full text of the articles (Table 1).

**Table 1:** Prevalence data collected from relevant articles for three main parasitic diseases in China

**Disease (n)**		**Count of articles**	**Survey population range**					**Epidemic data**		
			**0 ~ 1K (%)**	**1K ~ 10K (%)**	**10K ~ 100K (%)**	**100K ~ 1M (%)**	**1M ~ 10M (%)**	**Survey population**	**Positive serology rate**	**Average morbidity**
Malaria (154)	Survey for humans	154	13 (15.1%)	27 (31.4%)	24 (27.9%)	21 (24.4)	1 (1.1%)	110958	0.06%	0.00062% (0-0.2%)
	Survey for mosquito	6	0	2 (33.3%)	2 (33.3%)	2 (33.3%)	0	72776	-	0.009%
	Survey for animal(definitive host)	1	1 (100%)	0	0	0	0	328	3.4%	-
Schistosomisis (238)	Survey for human	238	53 (22.2%)	86 (36.1%)	61 (25.6%)	33 (13.8%)	5 (2.1%)	192061	14.2% (0–60.1%)	3.5 % (0–17.9%)
	Survey for Oncomelania hupensis	61	9 (14.7%)	18 (29.51%)	18 (29.51%)	13 (21.3%)	3 (4.9%)	57251	-	0.26% (0-1.2%)
	Survey for animal(definitive host)	75	23 (30.6%)	26 (34.6%)	18 (24.0%)	8 (10.6%)	0 (0.0%)	49773	-	3.81% (0–28.5%)
Echinococcosis (68)	Survey for human	68	21 (30.9%)	41 (60.3%)	6 (8.8%)	0	0	6022	20.8% (8.9-55.2%)	7.40% (0.15%-29.3%)
	Survey for pika, mice, livestock	40	21 (62.5%)	13 (35.0%)	1 (2.5%)	0	0	4016	-	24.9% (2.4%-54.9%)
	Survey for animal (definitive host)	18	14 (77.8%)	3 (16.7%)	1 (5.5%)	0	0	1609	39.2% (12.6-83.0%)	26.5% (1.0-70.4%)

#### Malaria

##### Prevalence

In 2011, there were 4479 malaria cases reported through the infectious diseases reporting system from 782 counties of 27 Provinces in P.R. China (total 2856 counties of 31 provinces). 68.9% of the reported cases were in Yunnan, Anhui, Jiangsu, Henan and Sichuan provinces [18].

**Figure 3:**
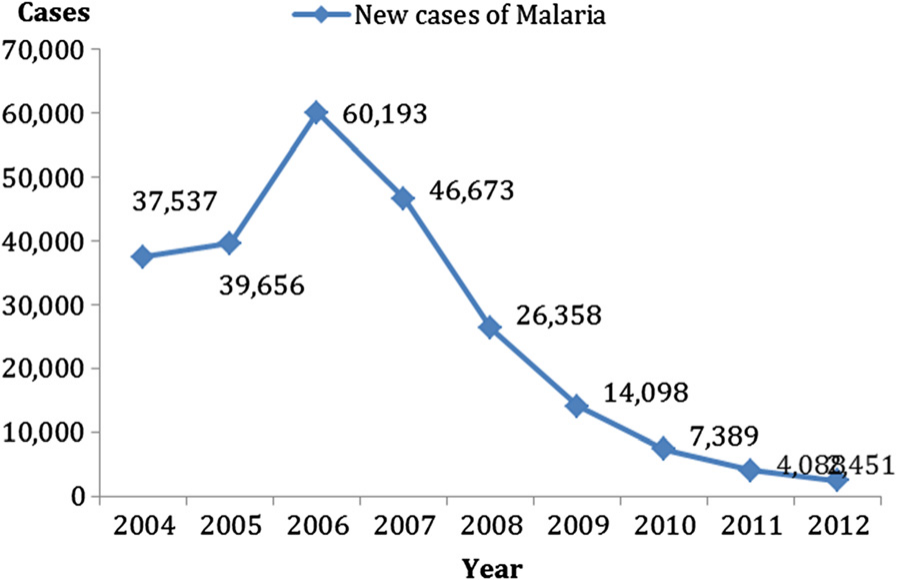
New Case Report of malaria in China (Year 2004–2012).

According to the Chinese Health Statistical Digest published by the Ministry of Health of P.R. China, the number of total malaria cases reported has declined significantly over the past ten years (Figure 3). Yet imported malaria cases began to dominate and became prevalent throughout the country after almost two years [19]. The imported malaria cases accounted for 66.4% of total malaria cases in 2011.

##### Gaps in eliminating malaria

When the goal for the national malaria control programme changes from control to elimination, the strategy on target population and control methods will need to be revised accordingly [20,21]. There are two gaps identified in eliminating malaria.

Gap 1: Lack of active surveillance in the public communities.

In the pre-elimination control stage, malaria carriers may be the main source of infection in the remaining epidemic area [22,23]. According to this research, only 5% malaria articles are relevant to active surveillance in the epidemic area. This condition was also identified from the national survey of malaria in 2010. In that year, the population tested for malaria in fever clinics was 7.1 million, while the field site survey population was only 0.14 million. Therefore, we recommend that more active surveillance activities be put in place to diagnose malaria carriers in suspicious residual epidemic sites in the future.

Gap 2: There is no appropriate technique to effectively evaluate elimination.

When indigenous malaria cases decreased sharply, the proportion of imported cases of falciparum malaria accounted that for all malaria cases increased from 1.5% in 2003 to 31.6% in 2011 [10,18,24-29]. However, there are still no articles relevant to the technique that can accurately distinguish imported cases from local cases [30]; this has also led to the problem of judging between new cases and recurring cases. In the later stage of malaria elimination, it is difficult to evaluate whether an area has really eliminated malaria or not. Thus, effective biomarkers urgently need to be developed in order to distinguish imported cases from local cases.

#### Schistosomiasis japonica

##### Prevalence

**Figure 4:**
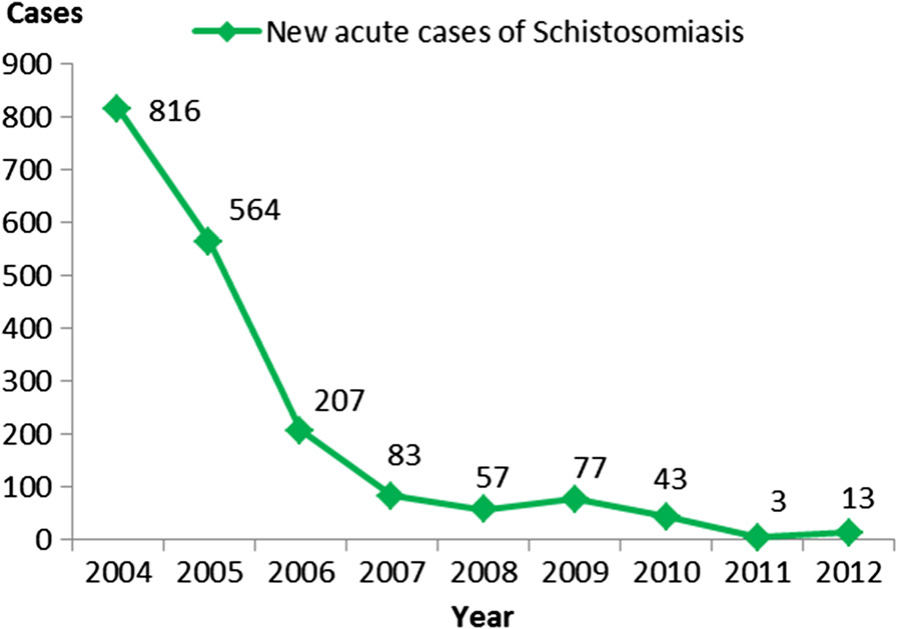
The number of acute cases of schistosomiasis japonica in China (Year 2004–2012).

In China, schistosomiasis japonica was epidemic throughout 12 provinces after liberation in the 1950s. By 1995 five provinces had blocked the transmission of *Schistosoma japonicum*[31]. However, transmissions still occurred in provinces along the Yangtze River and its southern areas in 2010, particularly in Hunan, Hubei, Jiangxi, Anhui and Jiangsu, and in the mountainous and hilly regions of Sichuan and Yunnan provinces. From 2004 to 2012, the number of acute cases of schistosomiasis japonica reported has dramatically declined from 816 to 13 [32-39] (Figure 4). However, there are still approximately 68 million individuals at risk [39].

In this study, 238 articles were judged relevant to epidemiology of *S*. *japonicum* in China. In Table 1, the average number of each survey article for human, *Oncomelania* sp snail and definitive host in animals are respectively 192061, 57251 and 49773. In these articles, most of the studies are reported from the 12 provinces mentioned above. To support the large amount of surveillance work that is needed to collect this information, China has thousands of professional staff responsible for the supervision and control of schistosomiasis japonica. For Hunan province, the Hunan Institute of Parasitic Diseases (HIPD) is an important guarantor of schistosomiasis control work throughout the province. The institute has over 400 professional staff and a hospital equipped with 300 beds for treating patients with schistosomiasis [40].

##### Gaps in controlling schistosomiasis japonica

Gap 1: Present policy cannot completely ensure the execution of an integrated intervention strategy for schistosomiasis control.

In China the national control programmes for schistosomiasis japonica is now at the transmission control stage. The goal at present is to decrease the schistosomiasis infection rate to 1% by 2015. Chinese authorities have also discussed the possibility of achieving elimination of schistosomiasis japonica by the year 2020. To realize these goals, a four-pronged approach has been investigated since 2008 [41,42]. The objective of the approach is to interrupt the environmental contamination of schistosome eggs as follows: First, replace buffaloes with tractors. Second, restrict marshland used for pasturing and encourage fenced cattle-farming. Third, improve sanitation facilities in houses. Fourth and last, provide toilets for mobile populations (e.g. fishermen). In general, the integrated measures for schistosomiasis japonica control with an emphasis on controlling the sources of infection have had strong effects [43-45]. However, in some less developed areas - particularly regions retaining traditional customs of using buffaloes for farming - considerable resistance has been faced for implementation because the strategy is contrary to the interests of the local economy [46-48]. Some marshland is still contaminated by infected cattle and many residents are under threat of schistosomiasis japonica [29].

Gap 2: Lack of effective tools for *Oncomelania sp*. snail control.

The habitat environment of the *Oncomelania* sp. snail in China has seen no significant change during the last 5 years [38,49,50]. *Oncomelania* sp. snails can proliferate in many kinds of habitat. In 2010, the total habitat area with snails was about 1608.7 million M^2^, presenting an ambitious task for elimination by manpower [51]. Three factors boost the expansion of the infected *Oncomelania* sp. snail’s habitats. 1) Unpredictable flooding of the Yangtze River. 2) More than 40 species of mammals that can act as reservoir hosts. 3) Millions of migrating people including patients moving to non-endemic rural areas. Such trends may threaten those non-endemic areas [52-56]. For this reason, the control of schistosomiasis japonica is not steady and can easily see repeated outbreaks. A new *Oncomelania* sp. snail control technique should be developed, for example, to find its natural predator or develop a biotechnological method to induce infecundity [57].

#### Echinococcosis

##### Prevalence

**Figure 5:**
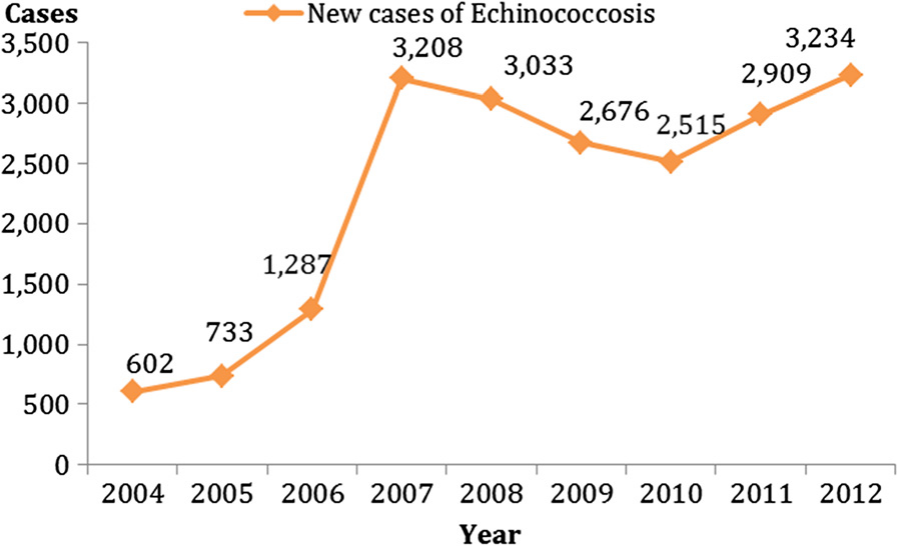
New Ccse reports of echinococcosis in P.R China (Year 2004–2012).

In China, new cases of echinococcosis have been increasing continuously since 2004 (Figure 5). The population at risk is approximately 66 million. The national survey of important parasitic diseases in 2004 showed that the average prevalence rate for populations in endemic areas was 1.08% [58]. From 2004 to 2008, 27 provinces out of 31 had reported cases of echinococcosis and 98.2% of the reported cases were in Inner Mongolia, Tibet, Gansu, Qinghai, Ningxia, Xinjiang and Sichuan province [58].

Echinococcosis is also a kind of zoonosis [59]. According to the “Prevention and treatment of echinococcosis Action Plan (2010–2015)”, the number of infected livestock in P. R. China amounts to about 50 million every year. Thus echinococcosis is one of the main reasons leading a large amount of herdsmen to poverty [60].

##### Gaps in control of echinococcosis

Gap 1: Low capacity for controlling echinococcosis

According to Chinese echinococcosis control policy, it is important to treat each dog in epidemic areas every month to control the main infection source [61,62]. The strategy is actually relatively straightforward, but the execution is very hard considering the enormous amount of dogs in these pasturing areas, which cover 40% of land in P.R. China [63-65]. In addition, most of the residents in these areas lack the corresponding prevention knowledge and are reluctant to change their living habits, such as feeding flesh viscera of sheep to dogs and touching dog fur [66-69].

Gap 2: Manpower restrictions in field survey.

According to the studies of echinococcosis in relevant papers, the total and average survey population (6,022) is far less than for the other two diseases (192,061 and 110,958). A low population density in epidemic areas, along with manpower restrictions are the main reasons for this situation occurring. It is imperative to train a larger number of echinococcosis researchers and improve their remuneration for these epidemic areas.

Gap 3: Not enough support for hygiene and sanitation in high transmission areas.

In high-transmission regions, the hygiene and sanitation conditions are usually unsatisfactory [70,71]. Most of these areas lack access to tap water; households collect water several times a day from contaminated lakes or rivers for personal use and drinking without boiling [72,73]. In addition the limited supply of water is not sufficient enough for washing hands [72,73]. These risk factors are hard to change for certain living environments and industries, especially in poor domestic economic situations.

### Gaps between diagnostic/drug development and population need for treatment

Relevant data has been collected and summarized to find the research gaps in diagnostic/drug development in the three diseases (Tables 2 and 3).

**Table 2:** Diagnosis data collected from relevant articles for three main parasitic diseases in China

**Disease**		**Immunodiagnosis method Research in field survey**				**Development of immunodiagnosis kit**					
**Disease**	**Total**	**Type (n)**	**Sensitivity**	**Specificity**	**Population**	**Total**	**Leading research direction**	**Labwork**	**Human test (n)**	**Basic Science**	**Marketization**
Malaria	26*	GICA(9)	88.4-95.5%	95.7-100%	547	35	GICA (9)	16	19	11/35	24/35
		ICA(14)	74.2-98.6%	91.3-100%	938						
		PCR(10)	96.1-99.6%	98.8-100%	167						
		LAMP(2)	98.3-98.8%	100%	46						
Schistosomiasis	55*	COPT(3)	90.0-91.8%	95.3-97.4%	89	52	Candidate Recombination Protein Finding (28)	41	11	44/52	8/52
		DDIA(8)	75.3-97.1%	55.1-99.0%	755						
		DIGFA(9)	92.0-100%	92.2-97.5%	556						
		ELISA(25)	65.8-98.4%	51.7-100%	716						
		IHA(32)	69.6-97.3%	63.6-97.8%	743						
		PCR(4)	100%	100%	52						
		Others(6)	85.5-98.5%	76.3-97.0%	85						
Echinococcosis	9*	IHA(4)	87.1-92.7%	69.5-91.6%	315	24	DIGFA (6)	8	16	5/24	19/24
		DIGFA(2)	56.7-93.2%	79.1-95.0%	180						
		ELISA(8)	59.3%-91.2%	93.8-100%	289						
		Others(3)	32.5%-76.9%	41.3-81.6%	42						

Note: *More than one diagnosis method was used in one article.

Abbreviation: *COPT* Circum oval precipitating test, *DDIA* Dipstick dye immuno-assay, *DIGFA* Dot immunogold filtration assay, *ELISA* Enzyme-linked immunosorbent Assay, *IHA* Indirect hemagglutination assay, *ELIB* Enzyme-linked immuno-electrotransfer blot, *ICA* immunochromatographic, *GICA* Gold immunochromatography assay, *LAMP* Loop-medicated isothermal amplification.

**Table 3:** Chemotherapy data collected from relevant articles for three main parasitic diseases in China

**Disease**	**Article (n)**	**Clinical trial for MOH Recommended drug**			**Drug development**			
		**Drug’s name (n)**	**Cure rates (%)**	**Population**	**Labwork (n)**	**Clinical trial (n)**	**Basic science**	**Marketization**
Malaria	51*	Dihydroartemisinin (31)	70%-100%	104 (15–1205)	50	0	50/50	0/50
		Artemether (30)	79.8%-100%					
		Piperaquine (19)	80%-100%					
		Artesunate (18)	90.7%-100%					
		Chloroquine (9)	62.8%-100%					
		Primaquine (9)	85.5%-100%					
		Artemether-lumefantrine (1)	82.50%					
Schistosomiasis	22	Praziquantel (22)	74.7%-100%	221 (33–615)	42	0	42/42	0/42
Echinococcosis	11*	Albendazole emulsion (7)	57.1%-83.1%	95 (36–264)	11	9	11/20	9/20
		Albendazole tablet (6)	15.8%-31.3%	57 (24–108)				

* More than one kind of drug can be studied in one article.

#### Gaps in diagnostic development

Gap 1: Lack of malaria sensitive diagnostic tools for asymptomatic patients and patients at low levels of parasitemia.

There were 61 papers that introduced the diagnosis of malaria. The availability of rapid diagnostic tests has made it possible to improve and expand diagnostic testing for malaria. In field surveys, PCR and LAMP diagnostic tools have the highest sensitivity (96.1-99.6%) and specificity (98.8-100%), but high cost and new technical requirements are obstacles for wider use [74]. GICA as a diagnostic tool appears to be the popular area in research and development as it is easy to use and has a lower cost, but the level of sensitivity is not satisfactory (88%-95%). We concluded from articles that in China over 90% of cases are tested by blood smears. This indicates that the diagnostic kit currently available on the market has not met the requirements of epidemic areas. A new diagnosis kit is needed for *P. vivax* with two components during pre-elimination stage. Firstly, it should have a high accuracy for low parasitemia density patients (both sensitivity and specificity should more than 95%). Secondly, it needs to detect G6PD deficiency (Glucose-6-phosphate dehydrogenase deficiency) because antimalarial drugs as primaquine, pamaquine, and chloroquine can cause acute haemolysis in people with G6PD deficiency [75].

Gap 2: Lack of a more sensitive and cheaper diagnostic test for *S. japonicum* infection of large population samples.

There were 107 papers introducing the diagnosis of schistosomiasis japonica. ELISA and IHA are the most common methods used on field sites. However, DIGFA has proved to have better sensitivity and accuracy. Therefore it is anticipated to be the future candidate used in large-scale surveys. In the research and development area, researchers focus on candidate-finding in recombination protein. This tendency is supported by the achievement of whole-genome sequencing of *S. japonicum* in China.

More than 80% of field surveys in China use immunodiagnostic kits combined with faecal examination. Antibody detection is very useful in screening for large-scale endemic surveys, in order to quickly acquire the baseline infection rate of schistosomiasis japonica. Faecal examination is recommended for determining the character that antibodies persist after parasitological cure. Moreover, microscopy may also miss infections, especially for those who are only moderately infected and in areas of low transmission [76-78]. Since an enormous amount of samples are to be identified for schistosomiasis elimination in the near future, the ideal diagnosis method should be more sensitive and significantly cheaper than any of the presently available methods.

Gap 3: Lack of sensitive test for early diagnosis of echinococcosis.

In China, ultrasonography is the most frequently used test for echinococcosis diagnosis [79,80]. Immunodiagnostic tests are also widely used in fieldwork. The kits used most widely are the ELISA-based serological tests, using either an *Echinococcus granulosus* hydatid cyst fluid antigen or an *E. multilocularis* crude vesicular fluid for primary screening. The sensitivity for hepatic cases of CE (*Cystic echinococcosis*) ranges from 85% to 98%. For AE (*Alveolar echinococcosis*), the use of purified or recombinant *E. multilocularis* antigens exhibit high diagnostic sensitivities ranging from between 91% and 100%, with overall specificities of 98%–100% [81]. However, for echinococcosis formed in other organs except for liver, the specificities are less than 50%. DIGFA seems to be the current area of interest for research and development.

In general, more imaging procedures should be used in echinococcosis epidemic areas to diagnose patients. For Immunodiagnostic tests, the gap is to develop new serological tests with higher sensitivity and specificity (>95%) than those based on the use of hydatid fluid and then bring them to a world standard. The resulting drug should also be straightforward and cheap to produce, with the capacity to diagnose early stage echinococcosis and allow for early treatment.

#### Gaps in drug development

Gap 1: Lack of safe drugs for both mass production and active again hypnozoites of *P. vivax*.

Antimalarial drugs are essential in moving from control to elimination [82]. A 8-day therapeutic schedule by using chloroquine plus primaquine for *P. vivax* has now replaced the old schedule for 21-days and 14-days in China. The patient cure rates for 8-days chemotherapy is 96.9%-100%. Also an artemisinin-based combination therapy is widely used in China to treat *P. Falciparum* with cure rate of 82.4%-100%. Still, no new drug has been successfully developed in the past 10 years in China.

*P.vivax* was detected in more than 99% of local malaria cases diagnosed microscopically. A characteristic of *P. vivax* infections is relapses originating from hypnozoites. There is a need for a radical cure to abate the source of infection during the course of malaria elimination [83]. New drugs are to be developed for hypnozoites and should be safe for patients with G6PD deficiency.

Gap 2: The public needs a new drug for prophylactic treatment of schistosomiasis.

Praziquantel is the sole drug for treatment and morbidity control of schistosomiasis japonica. Praziquantel is safe, cheap and effective against adult worms. In China, cure rates of up to 85–95.3% have been achieved but complete cures (100%) are seldom. Praziquantel does not prevent reinfection because it’s effects only last a few hours and it cannot kill immature worms [84]. Artemether was proved to kill immature worms over the first 21 days post inoculation in laboratory [85,86], yet there has been no further research to support its application and dissemination in large-scale chemotherapy combined with praziquantel for anti-schistosoma japonicum.

Many animal experiments have been conducted using mefloquine for anti-schistosomiasis [87-89]. The results indicate that mefloquine exhibits an extensive and severe damage both to juvenile and adult *S. japonicum* harbored in mice. Nevertheless, this is still a long way from field application when there is a public need also for a new drug in addition to praziquantel. Firstly, the new drug is required to be cheap, safe and effective so that it can be used for mass drug administration. Secondly, it should not produce the cross-resistance with praziquantel. Thirdly, it will preferably be effective against both juvenile and adult worms.

Gap 3: Lack of a more effective drug for a short-term treatment of echinococcosis.

In China, more than 94% echinococcosis patients were treated by chemotherapy, with only 6% of patients requiring an operation. Changing the dosage forms of albendazole tablets is the main achievement in echinococcosis chemotherapy in China [90,91]. Both albendazole emulsion and liposomal albendazole have proved more effective than albendazole tablet for *E. granulosus*[92-94]. However, there are shortages in the current chemotherapy approach as it is a long course of treatment (at least 3 months lifelong) and has relative low cure rates (8.2%-74.5%) [94-96]. An adverse effect rate of more than 20% is another main reason for patients to give up their medicine course [97,98]. Therefore, a new more efficient drug urgently needs to be developed.

### Gaps identified by consultations

The minutes of four consultation meetings were extracted into a list of the most important gaps presented by experts. This list in addition to the full text of included articles provides further detail to our study (See Table 4).

**Table 4:** Gaps of control capacity and diagnostic/drug development presented in total 4 consultation meetings

**Diseases**	**No.**	**Gap from consultation**
Malaria	1	Need new diagnosis method for asymptomatic patients
	2	Need innovative and safer drugs
	3	Lack of techniques to distinguish abroad-imported cases from indigenous cases
	4	Difficulty in finding asymptomatic patients
	5	Need new surveillance system for pre-elimination period
	6	More focus on the continuously increasing imported cases
Schistosomiasis japonica	1	Need higher sensitive antigen testing
	2	Need new drug which can prevent reinfection
	3	Need new molluscicide with less negative impact on the environment and biodiversity
	4	Difficulty in testing for a moving population
	5	Need innovative surveillance-response approaches system
	6	Difficulty in controlling for more than 40 hosts
Echinococcosis	1	Need effective drug for short-term treatment
	2	Need financial support for hygiene and sanitation in epidemic areas
	3	Need long-acting antihelmintic implants or drugs to control dog infection
	4	Need effective vaccine for people in epidemic areas
	5	Need more sensitive serological test for field survey
	6	Control of the wildlife transmission cycles of E multilocularis is difficult

## Discussion

The choice of scoping framework in the article through gap identification is based on our previous experience of scoping studies. The scoping review has centered on the identification and development of key research questions. The questions concern location, screening and selection of relevant publications; publication selection; data extraction; collating, summarizing and reporting the results. The resulting research gaps reported in our study also relied on two main sources. First, the literature review from which we extracted the papers to use and then summarized information from selected papers. Second, the gaps extracted in consultation. Overall we have identified, summarized and reported on about 13 research gaps in this paper. Experts in this study then gave corresponding advice on how to overcome gaps (See Table 5).

**Table 5:** Recommendations for overcome gaps of control capacity and diagnostic/drug development reported in this article

**Diseases**	**Research area of gaps**	**Specific gaps**	**Recommendation for overcome gaps**
Malaria	Epidemic control	Need more active surveillance for early detection	More precise prediction by statistical model and geographical information systems (GIS). Set up active surveillance and response system to prevent reestablishment of transmission.
		No appropriate techniques to evaluate elimination	Set up clonal germplasm repository for plasmodium and its vectors. Find specific biomarker to distinguish different geographic strain.
	Diagnosis	Need new diagnosis method for asymptomatic patient	Find new candidate biomarkers. Quality sampling for diagnostic kits before large-scale testing in field.
	Chemotherapy	Need innovative and safer drugs	Accelerate the validation and standardization for candidate drugs. Strengthen international cooperation in new drug development.
Schistosomiasis japonica	Epidemic control	Need effective tools for *Oncomelania sp*. snail control	Develop diagnostic assays for the large-scale screening of specific snails. Use GIS for prediction of snail distribution. Develop new snail interventions.
		Need policy support to protect the execution of control strategy	Coordinate by local government. Give full administration enforcement rights to the Chinese Center for Disease Control and Prevention.
	Diagnosis	Need more sensitive and cheaper diagnostic test for large population sampling.	Find new candidate biomarkers. Focus on innovation of immune complex dissociation procedure to increase the sensitivity of detection.
	Chemotherapy	Need new drugs in addition to praziquantel.	Accelerate the validation and standardization for candidate drugs. Strengthen international cooperation in new drug development.
Echinococcosis	Epidemic control	The execution for control echinococcosis is not satisfying.	Coordinate by local government. Give proper administration enforcement right to the Chinese Center for Disease Control and Prevention.
		Manpower restrictions in echinococcosis control	Larger personnel force for echinococcosis control. More professional training for echinococcosis control.
		Not enough support for hygiene and sanitation in epidemic areas.	Government increase in financial support on hygiene and sanitation improvement in these areas. Strengthen health education on echinococcosis prevention.
	Diagnosis	Need more sensitive test for early diagnosis	Undertake a comparative study of all the available antibody detection to form guidelines for large-scale scanning. Find new candidate biomarkers.
	Chemotherapy	Need more effective drug for short-term treatment	Accelerate the validation and standardization for candidate drugs. Focus to develop new form of benzimidazole to improve its bioavailability. Strengthen international cooperation in new drug development.

In this research, we endorse a consultation element to enhance a scoping review study. Choosing this route can enhance the veracity of final gap analysis and also ensure that the results more practically applicable. For instance, in the consultation meetings, experts presented the idea that P.R China needed a new technique to evaluate malaria elimination. Since P. R. China announced the objective to eliminate malaria only two years ago, few papers mentioned this gap. If we used a traditional systematic review, we may have not identified more immediate and forward-looking research gaps. Although such an element may be considered an ‘optional extra’ for scoping review, the consultation exercise did indeed provide ‘added value’ to the literature review. The limit of this research is in the deficiency of vaccine analysis. We do not have any professional team members working on the vaccine area. Our concern was in giving a fair evaluation on the part of vaccine research and therefore we have not incorporated vaccine research into the theme of the study.

When comparing the research priorities among the three diseases, malaria, schistosomiasis and echinococcosis, in aspects of diagnostics and drugs the major difference was found in two areas. One is the requirements of sensitivity and specificity in diagnosis, and the other is effectiveness of drug treatment, due to the different stage of the diseases control programme. For instance, for the pre-elimination stage of malaria, higher sensitivity and specificity diagnostics is needed both in individual and in population diagnosis [99]. At the same time, the tools used in the certification of malaria elimination, also needs to be further developed [100]. This is a concern as to the fact that innovative drugs or drug combinations are given a higher priority to reduce the risk of drug resistance [101,102]. For the transmission control stage of schistosomiasis, it is necessary for high sensitivity tools to screen the at risk population, in order to guide the MDA or selective chemotherapy based on different endemicity levels and further reduce transmission risks [5,77,78]. New drugs need to be developed for prophylactic treatment to effectively for target all stages of the parasites [2]. For the morbidity or infection control stage of echinococcosis, it is necessary to develop the early diagnostics, for individual and population uses [103]. Effective drugs with a short-time chemotherapy also requires development [3]. Both early diagnostics and short-time schemes for the effective treatment will contribute significantly to the reduce burden of disease [104]. Therefore, this comparative approach provides a clear indication of the diagnostic drugs that are necessary to be used in the different stages of the control programme.

The major problem has been that investment into the national control or elimination programmes has fallen after the burden of disease. We suggest that sustained investment in the development of diagnostics and drugs is required. This needs to be well budgeted for at all stages, with the argument put to policy makers that a marginal effect applies in the different stages of the national control programme, whether it be from morbidity or infection control to elimination.

## Conclusions

Today, although some areas of research on schistosomiasis japonica, malaria and echinococcosis have been neglected, they are now attracting high levels of concern from many nations, including P.R China. The government is providing sustained financial and technical support underpinned by key control targets. Thus we believe these three diseases can eventually be controlled or eliminated in mainland China following years of sustained efforts.

### Endnote

^a^In Medline database, articles were searched by: (“disease name”[MeSH Terms] OR “disease name”[All Fields]) AND (“china”[MeSH Terms] OR “china”[All Fields]) AND (“2007/06/05”[PDat]: “2012/06/05”[PDat] AND English [lang]). In Chinese Wanfangdata, articles were searched by (“disease name”[MeSH Terms] or “disease name “[All Fields]) and (“therapy” [MeSH Terms] or “treatment” or “drug-resistance” [MeSH Terms] or “adverse event” [MeSH Terms] or “diagnosis” [MeSH Terms] or “examination” or “test” or “epidemiology” [MeSH Terms] or “survey” or “surveillance” or “investigation” or “monitoring from June 2007 to June 2012.

## Competing interests

The authors declare that they have no conflicting interests.

## Authors’ contributions

QZ conceived the study, carried out data collection and analysis and drafted the manuscript. HBZ conceived the project and revised the manuscript. SV revised the manuscript and provided intellectual input to the interpretation of the findings. BJ, LLX, CSL, LLH, LPD and NBW conceived the project and carried out data collection and analysis. SZL, ZGX, WPW, WH conceived the project and revised the manuscript. HBZ mainly conceived the project and revised the manuscript. All authors read and approved the final manuscript.

## Supplementary Material

Additional file 1 Multilingual abstracts in the six official working languages of the United Nations. Click here for file
