# Supplementary material for: Research gaps for three main tropical diseases in the People’s Republic of China
Source: Infect Dis Poverty. 2013 Jul 29;2:15. doi: 10.1186/2049-9957-2-15 (PMC3751495; doi:10.1186/2049-9957-2-15)
Supplement: Additional file 1 — Multilingual abstracts in the six official working languages of the United Nations. [file 2049-9957-2-15-S1.pdf]

Translation of the abstract into the six official working languages of the United Nations

الفجوات البحثية لثلاثة أمراض مدارية رئيسية في جمهورية الصين الشعبية

تشى تشنغ - سمانثا فندرسلوت - بن جيانغ - لى لى شو - تسونغ شان ليو – لي لي هيو - لى بينغ دوان - نينغ بو وو - شي تشو لى – زي جي شيا - وى بينغ وو - وي هو - هاو بينغ تشانغ.

ملخص

هذا العرض الفحصي يحلل الفجوات البحثية لثلاثة أمراض: داء البِلْهارْسِيَّا اليابانِيَّة – داء الملاريا - داءُ المُشْوكات.

بناءً على البيانات المتوفرة لدى جمهورية الصين الشعبية، نسلط الضوء هنا على الفجوات بين قدرة التحكم ومستويات الانتشار – بين تطور الأدوية والتشخيص من جانب وبين احتياج السكان للعلاج في مراحل مختلفة من برنامج المكافحة الوطني من جانب آخر.

بعد مراجعة أدبيات 848 دراسة أصلية واستشارة خبراءٍ في هذا المجال، تم تحديد الفجوات كالآتي:

أولاً، الفجوات البحثية لداء الملاريا يشمل: (أ) نقص في الاختبارات الفعّالة في المجتمع العام وعدم وجود تقنيات ملائمة لتقييم التخلص من المرض. (ب) نقص في أدوات تشخيص دقيقة لمرضى بدون أعراض. (ج) نقص في الأدوية الآمنة للاستخدام الشامل.

ثانياً، الفجوات البحثية لداء البلهارسيا يشمل: (أ) سياسة متعارضة لتفعيل ستراتيجية المكافحة الشاملة لداء الملاريا.

(ب) نقص في أدوات فعالة لمكافحة القواقع ("القواتم" نوع من القواقع). (ج) نقص في اختبارات تشخيصية أكثر دقةوأقل كلفة لعينات لمجموعة كبيرة من السكان. (د) نقص في الأدوية الجديدة إلى جانب دواء البرازيكوانتيل.

ثالثاً، الفجوات البحثية لداء المشوكات يشمل: (أ) القدرة المنخفضة في مجال دراسات الوبائيات. (ب) نقص في دراسات تحسين الصرف الصحي في الأماكن الموبوءة. (ج) نقص في اختبارات دقيقة للتشخيص المبكر. (د)نقص في الأدوية الفعّالة للعلاج القصير الأمد.

في نهاية المطاف نعتقد أنه بالامكان القضاء على هذه الأمراض الثلاث في الصين في وقت قصير اذا ما استطعنا أن نقلص كل الفجوات البحثية المذكورة.

Translated from English version into Arabic by Catherine Hanna, through

TRANSLATORS WITHOUT BORDERS

三种中国主要热带疾病的研究空白

郑琪, Samantha Vanderslott, 姜斌, 徐莉莉, 刘丛珊, 霍乐乐, 段李平, 吴宁波, 李石柱, 夏志贵, 伍卫平, 胡薇, 张皓冰

摘要

这篇勘域综述基于中华人民共和国的现有数据，分析了三种疾病（日本血吸虫病、疟疾和棘球蚴病）中存在的研究空白。我们突出了在疾病控制能力和实际流行水平之间研究空白，以及在不同流行阶段全体居民对治疗的需求同诊断/药物研究的实际现状之间的研究空白。在回顾了 848 篇研究文献和开展专家咨询工作后，确定了以下研究空白。首先，疟疾中包括的研究空白有：1）缺乏对公众人群中疟疾患者的主动调查以及缺乏相应评估消除疟疾的技术手段。2）缺乏敏感的诊断工具用于疟疾无症状患者 3）缺乏安全的药物用于大规模人群。第二，在血吸虫病中的研究空白包括：1）血吸虫病的综合防治策略缺乏适用的法律法规支持其执行。2）缺乏有效控制钉螺的手段 3）缺乏更敏感并且廉价的检测手段用于大规模人群调查 4）除吡喹酮外缺乏有效新药。第三，在棘球蚴病中的研究空白包括：1）现场流行病学调查中的人群数量少，缺乏代表性 2）在棘球蚴病流行区缺乏关于卫生设施改善方面的研究 3）缺乏有效的早期诊断方法 4）缺乏疗程较短的有效药物。我们相信如果这些研究空白在较短时间内得到填补，这三种疾病最终会在中国大陆被消除。

Translated from English version into Chinese by Zheng Qi, through

Lacunes en matière de recherche sur trois principales maladies tropicales en République populaire de Chine

Qi Zheng, Samantha Vanderslott, Bin Jiang, Li-Li Xu, Cong-Shan Liu, Le-Le Huo, Li-Ping Duan, Ning-Bo Wu, Shi-Zhu Li, Zhi-Gui Xia, Wei-Ping Wu, Wei Hu et Hao-Bing Zhang

Résumé

Cet examen de la portée analyse les lacunes en matière de recherche sur trois maladies : la schistosomiase intestinale asiatique, le paludisme et l'échinococcose. En nous basant sur des données disponibles en République populaire de Chine (RPC), nous soulignons les écarts entre la capacité de contrôle et les taux de prévalence, et entre le diagnostic/développement des médicaments et les besoins de traitement de la population à différents stades du programme national de contrôle. Après avoir examiné 848 études originales et consulté des experts dans ce domaine, les lacunes ont été identifiées comme suit. Premièrement, les lacunes en matière de recherche sur le paludisme incluent : (i) l’insuffisance de tests actifs dans la collectivité publique et l’absence de techniques appropriées pour évaluer l’élimination, (ii) le manque d’outils de diagnostic sensibles pour les patients asymptomatiques et (iii) le manque de médicaments sûrs pour l’administration en masse. Deuxièmement, les lacunes en matière de recherché sur la schistosomiase incluent : (i) une politique inadéquate dans la mise en œuvre d’une stratégie de contrôle intégrée contre la schistosomiase, (ii) le manque d’outils efficaces contre *l’Oncomelania*, en particulier le contrôle des escargots, (iii) l’absence d’un test de diagnostic plus sensible et meilleur marché pour de grands échantillons de population et (iv) le manque de nouveaux médicaments outre le praziquantel. Troisièmement, les lacunes dans la recherche sur l’échinococcose incluent : (i) de faibles capacités dans le domaine des études épidémiologiques, (ii) le manque d’études consacrées à l’amélioration de l’assainissement dans les zones d’épidémie, (iii) l’absence d’un test sensible pour le diagnostic précoce et (iv) le manque de médicaments plus efficaces pour le traitement à court terme. Nous croyons que ces trois maladies finiront par être éradiquées en Chine continentale si toutes les lacunes en matière de recherche sont comblées en un court laps de temps.

Translated from English version into French by Laetitia Michel, through

TRANSLATORS
WITHOUT BORDERS

К вопросу о проблемах изучения трех наиболее распространенных тропических заболеваний в Китайской Народной Республике

Кви Женг, Саманта Вандерслотт, Вин Джианг, Ли-Ли Ксу, Конг-Шан Лиу, Ле-Ле Хуо, Ли-Пинг Дуан, Нанг-Бо Ву, Ши-Жу Ли, Жи-Хуи Ксиа, Вей-Пинг Ву, Вей Ху и Хао-Бинг

Аннотация

Данная аннотация представляет собой анализ проблем, возникающих при изучении трех заболеваний: японского шистосоматоза, малярии и эхинококкоза. На основе имеющихся данных, доступных для ознакомления в Китайской Народной Республике (КНР), мы выделяем следующие проблемы: 1) возможность контроля и уровни распространения, 2) разработка диагностики/препарата и количество заболевших, нуждающихся в лечении, на разных этапах программы национального контроля. После анализа научной документации первичных исследований (в совокупности 848) и консультаций с экспертами в данной области, были определены следующие проблемы изучения. Во-первых, проблемами, возникающими при изучении малярии, являются следующие: (i) отсутствие проведения плановых периодических проб у населения и отсутствие соответствующих методик по оценке ликвидации, (ii) нехватка точных диагностических инструментов для диагностирования бессимптомного протекания заболевания и (iii) нехватка медикаментов, безопасных для населения. Во-вторых, в связи с изучением шистосоматоза, возникают проблемы следующего характера: (i) непоследовательная политика по всеобъемлющему контролю шистосоматоза, (ii) нехватка эффективных средств по контролю за распространением улиток рода *Онкомелания*, (iii) отсутствие более точной и менее затратной диагностической процедуры для анализа образцов, взятых у большого количества населения, а также (iv) нехватка новых медикаментов, помимо празиквантела. В-третьих, при изучении эхинококкоза возникают такие проблемы как: (i) недостаточный объем полевых эпидемиологических исследований, (ii) отсутствие исследований по улучшению санитарных условий в зонах эпидемии, (iii) нехватка точных инструментов для ранней диагностики, а также (iv) нехватка более эффективных медикаментов для быстрого лечения. Мы верим, что эти три заболевания, в итоге, могут быть ликвидированы на территории Китая, при условии, что все вышеперечисленные проблемы будут

устранены в ближайшее время.

Translated from English version into Russian by Halyna Maksymiv, through

TRANSLATORS
WITHOUT BORDERS

Brechas en la investigación de tres importantes enfermedades tropicales en la República Popular China

Qi Zheng, Samantha Vanderslott, Bin Jiang, Li-Li Xu, Cong-Shan Liu, Le-Le Huo, Li-Ping Duan, Ning-Bo Wu, Shi-Zhu Li, Zhi-Gui Xia, Wei-Ping Wu, Wei Hu and Hao-Bing Zhang

Resumen

Esta revisión general analiza las brechas en la investigación de tres enfermedades: esquistosomiasis japónica, malaria y echinococcosis. Tomando como referencia los datos disponibles en la República Popular China (R.P. China), hemos subrayado las brechas existentes entre la capacidad de control y los niveles de prevalencia, y entre el desarrollo de diagnósticos/fármacos y la necesidad, por parte de la población, de tratamiento en diferentes etapas del programa de control nacional. Tras revisar 848 estudios originales y haber consultado a expertos en este campo, se han identificado las brechas señaladas a continuación. Primero, entre las brechas para la investigación sobre la malaria se encuentran: (i) déficit de pruebas médicas en la comunidad pública y falta de técnicas apropiadas para evaluar la eliminación, (ii) carencia de herramientas de diagnóstico capaces de detectar a pacientes asintomáticos y (iii) carencia de medicamentos seguros para su administración de forma generalizada. Segundo, entre las brechas para la investigación sobre la esquistosomiasis se encuentran: (i) política incongruente para la implementación de una estrategia de control integrada de la esquistosomiasis, (ii) carencia de herramientas eficaces para el control del caracol *Oncomelania* sp., (iii) carencia de una prueba diagnóstica más precisa y económica para grandes muestras de población y (iv) carencia de nuevos medicamentos además de praziquantel. Tercero, entre las brechas para la investigación sobre la echinococcosis se encuentran: (i) baja capacidad en estudios epidemiológicos sobre el terreno, (ii) carencia de estudios sobre mejoras de tipo sanitario en áreas epidémicas, (iii) carencia de pruebas de sensibilidad capaces de realizar diagnósticos en fase temprana y (iv) carencia de medicamentos más eficaces para tratamientos a corto plazo. Creemos que estas tres enfermedades se pueden erradicar por fin en la China continental si se cierran todas las brechas en la investigación en un corto período de tiempo.

Translated from English version into Spanish by Sergiman, through

TRANSLATORS
WITHOUT BORDERS
